# A pilot clinical study of Δ^9^-tetrahydrocannabinol in patients with recurrent glioblastoma multiforme

**DOI:** 10.1038/sj.bjc.6603236

**Published:** 2006-06-27

**Authors:** M Guzmán, M J Duarte, C Blázquez, J Ravina, M C Rosa, I Galve-Roperh, C Sánchez, G Velasco, L González-Feria

**Affiliations:** 1 Department of Biochemistry and Molecular Biology I, School of Biology, Complutense University, Madrid 28040, Spain; 2 Department of Neurosurgery, Hospital Universitario de Canarias, La Laguna, Tenerife 38320, Spain

**Keywords:** cannabinoid, glioblastoma multiforme, pilot clinical study, antitumoral drug

## Abstract

Δ^9^-Tetrahydrocannabinol (THC) and other cannabinoids inhibit tumour growth and angiogenesis in animal models, so their potential application as antitumoral drugs has been suggested. However, the antitumoral effect of cannabinoids has never been tested in humans. Here we report the first clinical study aimed at assessing cannabinoid antitumoral action, specifically a pilot phase I trial in which nine patients with recurrent glioblastoma multiforme were administered THC intratumoraly. The patients had previously failed standard therapy (surgery and radiotherapy) and had clear evidence of tumour progression. The primary end point of the study was to determine the safety of intracranial THC administration. We also evaluated THC action on the length of survival and various tumour-cell parameters. A dose escalation regimen for THC administration was assessed. Cannabinoid delivery was safe and could be achieved without overt psychoactive effects. Median survival of the cohort from the beginning of cannabinoid administration was 24 weeks (95% confidence interval: 15–33). Δ^9^-Tetrahydrocannabinol inhibited tumour-cell proliferation *in vitro* and decreased tumour-cell Ki67 immunostaining when administered to two patients. The fair safety profile of THC, together with its possible antiproliferative action on tumour cells reported here and in other studies, may set the basis for future trials aimed at evaluating the potential antitumoral activity of cannabinoids.

The hemp plant *Cannabis sativa* L. produces approximately 60 unique compounds known as cannabinoids, of which Δ^9^-tetrahydrocannabinol (THC) is the most important owing to its high potency and abundance in cannabis (Gaoni and Mechoulam, 1964). Δ^9^-Tetrahydrocannabinol exerts a wide variety of biological effects by mimicking endogenous substances – the so-called endocannabinoids (Mechoulam and Hanus, 2000; Piomelli, 2003) – that bind to and activate specific cell surface receptors. So far, two cannabinoid-specific receptors have been cloned and characterised from mammalian tissues (Howlett *et al*, 2002): CB_1_, particularly abundant in the brain, and CB_2_, mainly expressed in the immune system. One of the most active areas of current research in the cannabinoid field is the study of the potential application of cannabinoids as therapeutic agents. Among these possible applications, cannabinoids have been known to exert palliative effects in cancer patients since the early 1970s. The best established of these effects is the inhibition of chemotherapy-induced nausea and vomiting. Today, capsules of THC and its synthetic analogue nabilone are approved in several countries for that purpose (Guzmán, 2003; Hall *et al*, 2005). Other potential palliative effects of cannabinoids in oncology – supported by phase III clinical trials – include appetite stimulation and pain inhibition (Guzmán, 2003; Hall *et al*, 2005). In addition, cannabinoids have been proposed as potential antitumoral agents owing to their ability to inhibit the growth and angiogenesis of various types of tumour xenografts in animal models (Munson *et al*, 1975; Guzmán, 2003). However, the antitumoral effect of cannabinoids has never been tested in humans.

One of the most devastating forms of cancer is glioblastoma multiforme (grade IV astrocytoma), the most frequent class of malignant primary brain tumours. Current standard therapeutic strategies for the treatment of glioblastoma multiforme (surgical resection and focal radiotherapy) are only palliative, and, as a consequence, survival after diagnosis is normally 6–12 months (Afra *et al*, 2002; Kleihues *et al*, 2002; Lonardi *et al*, 2005). A large number of chemotherapeutic agents (e.g. alkylating agents such as temozolomide and nitrosoureas such as carmustine) have also been tested, but no remarkable improvement on patient survival has been achieved as yet (Afra *et al*, 2002; Lonardi *et al*, 2005; Reardon *et al*, 2006). Likewise, although dendritic cell- and peptide-based immunotherapy strategies appear promising as a safe approach to induce an antitumour immune response (Yamanaka, 2006), no immunotherapy or gene therapy trial performed to date has been significantly successful. It is therefore essential to develop new therapeutic strategies for the management of glioblastoma multiforme to obtain significant clinical results. We have previously shown that cannabinoids inhibit the growth (Galve-Roperh *et al*, 2000; Sánchez *et al*, 2001) and angiogenesis (Blázquez *et al*, 2003, 2004) of gliomas in animal models. Remarkably, this antiproliferative effect seems to be selective for brain-tumour cells as the survival of normal brain cells (astrocytes (Gómez del Pulgar *et al*, 2002), oligodendrocytes (Molina-Holgado *et al*, 2002) and neurons (Mechoulam *et al*, 2002)) is unaffected or even favoured by cannabinoid challenge. On the basis of these preclinical findings, we have conducted a pilot clinical study aimed at assessing cannabinoid antitumoral action in patients with recurrent glioblastoma multiforme.

## PATIENTS AND METHODS

### Patients

Nine patients with glioblastoma multiforme were enroled. All patients had failed standard therapy, which included surgery and external-beam radiotherapy (60 Gy), had clear evidence of tumour progression on sequential magnetic resonance scanning, and had a minimum Karnofsky performance score (KPS) of 60 (i.e. ability to function independently). Two patients (Patients 2 and 5) had received adjuvant temozolomide chemotherapy (two cycles of 150–200 mg m^−2^ daily for 5 days; 28 days between the two cycles), but temozolomide administration had finished 24 weeks (Patient 2) or 8 weeks (Patient 5) before enrolment in the study. Pregnant patients and patients with systemic diseases or active infections were excluded. Additional requirements included acceptable haematological and hepatic function (glutamate : oxalacetate transaminase and glutamate : piruvate transaminase <2.5 × normal values, total bilirubin <1.5 × normal values, platelets >100 000 mm^−3^, neutrophils >1000 mm^−3^, haemoglobin >10 g dl^−1^) and no change in steroid administration protocol for at least 2 weeks. All patients provided written informed consent before entering the study. The protocol, progression and final report of the study were approved by the Clinical Trials and Ethics Committee of Hospital Universitario de Canarias and by the Spanish Ministry of Health.

### THC administration

THC was obtained from The Health Concept (Richelbach, Germany) and was kindly provided by Mr Alfredo Dupetit. Preparations contained >96.5% THC, <1.5% of its isomer Δ^8^-THC, <0.5% butyl-THC and <0.5% propyl-THC.

Patients underwent a surgical intervention aimed at resecting and creating a cavity in the recurrent tumour. Biopsies were taken and glioblastoma multiforme diagnosis was confirmed in all cases. The tip (approx. 5 cm) of a silastic infusion catheter (9.6 French; 3.2 mm diameter) was placed into the resection cavity. The infusion catheter was connected to a Nuport subclavicular subcutaneous reservoir. Each day an aliquot of the THC solution (100 mg ml^−1^ in ethanol) was dissolved in 30 ml of physiological saline solution supplemented with 0.5% (w v^−1^) human serum albumin, and the resulting solution was filtered and transferred to an opaque syringe. This process was performed at the Department of Pharmacy of the Hospital Universitario de Canarias. Owing to the high hydrophobicity of THC, we controlled by gas chromatography/mass spectrometry (see below) the actual concentration of THC in the final solution. The THC solution was administered to the patients for different times starting at days 3–6 after surgery at a rate of 0.3 ml min^−1^ with a syringe pump connected to the subcutaneous reservoir. In the case of Patients 1 and 2, who received THC for 30 and 26 days, respectively, biopsies were also taken after the THC-treatment period and various tumour-cell parameters were evaluated (see below).

### Patient monitoring

Patients underwent continuous physical, neurological, biochemical and haematological examinations as well as frequent magnetic resonance and computed tomography scans of the brain for the detection of evidence of toxicity (haemorrhage, oedema, injury) and monitoring of tumour progression. Magnetic resonance imaging evaluations (T1-weighted gadolinium enhancement axial images) measured enhanced tumour size, which is believed to represent the viable portion of the tumour.

### Determination of THC concentration

The plasma and urine concentration of THC was determined daily in Patients 1 and 2 during the first 7 days of administration by three different methods: a fluorescence polarisation immunoassay kit (AxSYM Cannabinoid Assay, Abbott, Abbott Park, IL, USA; detection limit, 50 ng ml^−1^), a cloned enzyme donor immunoassay kit (Microgenics CEDIA DAU Multi-Level THC, Microgenics, Pleasanton, CA, USA; detection limit, 50 ng ml^−1^) and gas chromatography/mass spectrometry (determinations performed at the Department of Toxicology, School of Medicine, Complutense University, Madrid, Spain; detection limit, 10 ng ml^−1^). These procedures also detected the major THC metabolites 11-hydroxy-THC and 11-nor-THC-9-carboxylic acid. In all the analyses performed in both plasma and urine, the concentrations of THC and its two metabolites were below the detection limits. Gas chromatography/mass spectrometry (determinations performed at the Institute of Toxicology, Ministry of Health, Tenerife) was also used to determine the actual concentrations of THC in the final solution inoculated to the patients.

### Tumour cell cultures

Tumour biopsies were digested with collagenase (type Ia, Sigma, St Louis, MO, USA) in Dulbecco's modified Eagle's medium (DMEM) at 37°C for 90 min and the supernatant was seeded in DMEM containing 15% foetal calf serum and 1 mM glutamine. Cells were kept in primary culture for about 2 weeks. Cells were subsequently seeded for the experiments, and 24 h before cannabinoid addition they were transferred to 0.5% serum DMEM. Cell viability was determined by Trypan blue exclusion. Apoptosis was determined by Hoechst 33258 staining and with a terminal deoxynucleotide transferase-mediated deoxy uridine triphosphates-biotin nick-end labelling (TUNEL) kit (Boehringer, Mannheim, Germany). THC as well as SR141716 and SR144528 (kindly given by Sanofi-Aventis, Montpellier, France) were prepared in dimethylsulphoxide (final concentration: 0.1–0.2% (v v^−1^)). Control incubations had the corresponding dimethylsulphoxide content. All determinations were performed in triplicate.

### Western blot

Tumour samples were homogenised and subjected to sodium dodecyl sulphate–polyacrylamide gel electrophoresis, and proteins were transferred from the gels onto polyvinylidene fluoride membranes. The blots were incubated with antibodies raised against residues 1–77 of the human CB_1_ receptor (1 : 1000; kindly given by Ken Mackie, University of Washington, Seattle, WA, USA) or residues 1–99 of the human CB_2_ receptor (1 : 1000; Affinity Bioreagents, Golden, CO, USA). *α*-Tubulin (1 : 4000, Sigma) was used as a loading control. Samples were finally subjected to luminography with an enhanced chemiluminiscence detection kit (Amersham Life Sciences, Arlington Heights, IL, USA). Densitometric analysis of the blots was performed with Multianalyst software (Bio-Rad Laboratories, Hercules, CA, USA).

### Confocal microscopy

Sections of formalin-fixed, paraffin-embedded tumour samples were stained with anti-CB_1_ receptor (1 : 500; kindly given by Ken Mackie), anti-CB_2_ receptor (1 : 500; Affinity Bioreagents), anti-Ki67 (Lab Vision, Fremont, CA, USA) or anti-CD31 (1 : 400; Cymbus Biotechnology, Hampshire, UK) antibodies as described (Blázquez *et al*, 2004). Slices were further incubated (1 h, room temperature, darkness) with a secondary antibody–Alexa Fluor 594 (1 : 400; Molecular Probes, Leiden, The Netherlands). Sections were mounted with Mowiol mounting medium (Merck, Darmstadt, Germany) containing YOYO-1 iodide (1 : 1000; Molecular Probes, Leiden, The Netherlands) to stain cell nuclei. Ten to fifteen fields of 4–6 sections were analysed per tumour. Morphometric analysis was performed with Metamorph-Offline software (Universal Imaging, Downingtown, PA, USA).

### Statistics

**Figure 1:**
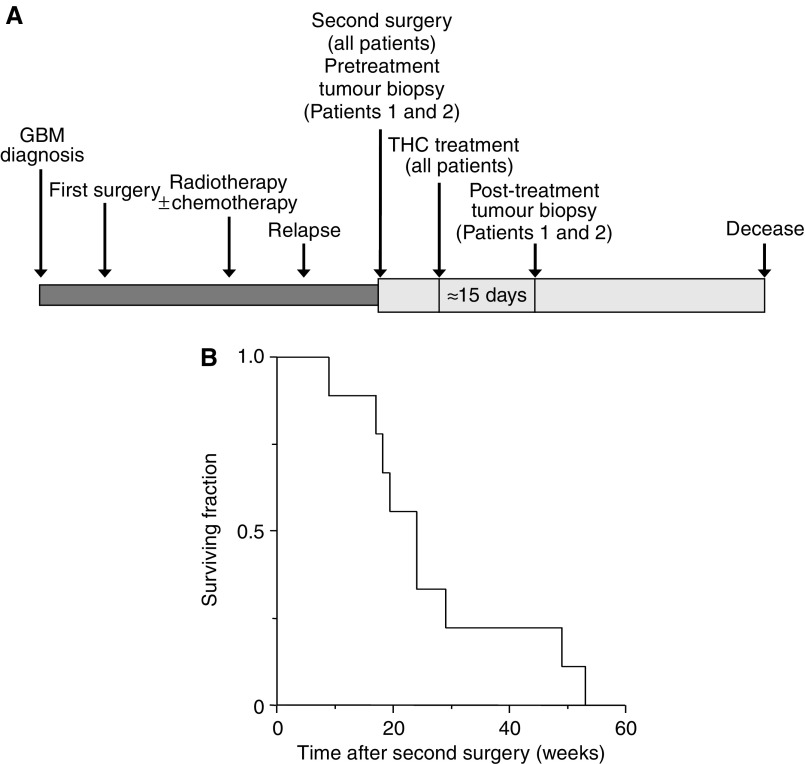
Effect of THC administration on overall survival. (**A**) Schematic diagram of the clinical protocol. See text for further details. (**B**) Kaplan–Meier survival curve of the cohort of patients from the surgical operation of tumour relapse. For comparison with survival upon administration of standard chemotherapeutic drugs such as temozolomide and carmustine, see Dinnes *et al* (2002) and Brem *et al* (1995), respectively.

Survival was calculated as the median (95% confidence interval (CI)) survival time of the cohort of patients from the surgical operation of tumour relapse, and was represented as a Kaplan–Meier curve (Figure 1B). Data on tumour-cell parameters (Figure 3) are given as mean±s.d. and were analysed by analysis of variance with a *post hoc* Student–Neuman–Keuls test.

## RESULTS

### Patients

Nine patients (four men, five women) were enroled in the study between March 2002 and November 2003 (Table 1). The cohort had a mean age of 55 years and a moderately altered physical performance (mean KPS: 81; most frequent symptoms on enrolment: cephalalgia, alterations in higher cerebral functions, long tract signs and epilepsy in six out of nine, four out of nine, eight out of nine and three out of nine patients, respectively). The recurrent tumours had medium–large size (mean estimated volume of active tumour: 64 cm^3^) and had appeared after a period expected for average glioblastoma multiforme progression (mean interval between first and second surgery: 42 weeks). The cohort, although small, was therefore considered representative of recurrent glioblastoma multiforme routinely found in the clinical practice, which would make the study unbiased towards patients with better prognosis. The primary end point of the study was to determine the safety of intracranial THC administration. We also assessed THC action on the length of survival and various tumour-cell parameters. The clinical protocol is summarised in Figure 1A.

### Safety of the treatment

Patients were entered one by one in order to ascertain a dose escalation regimen for THC administration based on the appearance of psychoactive side effects. In Patient 1, the initial daily dose of THC was 20 *μ*g, which was progressively increased for 4 days until 100 *μ*g, with which a very mild episode of euphoria appeared. This effect was transient and difficult to interpret, as it never repeated. The daily dose was subsequently set at 60–80 *μ*g and no further side effects appeared anymore during the first cycle (19 days, 0.98 mg total THC) or the second cycle (11 days, 0.48 mg total THC). A similar approach was used to define the administration pattern of THC to other patients and no significant psychoactive effects were evident, except for Patient 8, who had a mild and transient episode of bulimia, hypothermia and euphoria. Overall, the initial dose of THC administered to the patients was 20–40 *μ*g at day 1, increasing progressively for 2–5 days up to 80–180 *μ*g day^−1^. The median duration of an administration cycle was 10 days. Some patients received more than one THC cycle (Table 1), and so the median duration of total THC administration was 15 days (Figure 1A). Of interest, no significant alterations in physical, neurological, biochemical and haematological parameters could be ascribed to THC in any of the patients. All patients experienced cerebral oedema during the study, as is typical for postoperative craniotomy, and were treated with corticosteroids. There was no apparent effect of THC on steroid requirement.

### Progression and survival

Median survival from the surgical operation of tumour relapse was 24 weeks (95% CI: 15–33). Two of the patients (3 and 8) survived for approximately 1 year (Table 1, Figure 1B).

**Figure 2:**
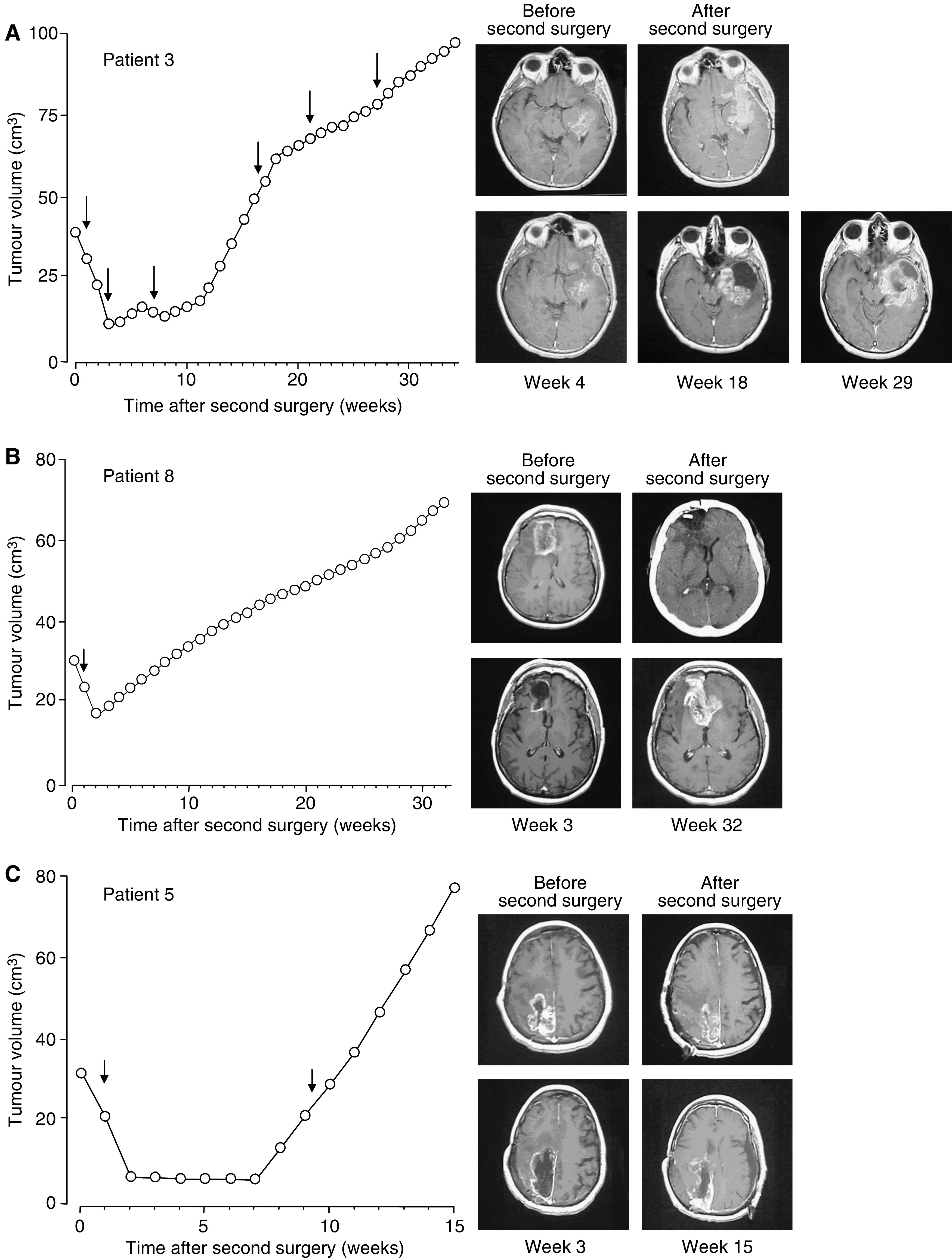
Effect of THC administration on tumour growth. Tumour growth plots and gadolinium-enhanced T1-weighted magnetic resonance scans after the second surgery in three patients. Arrows indicate the THC administration cycles. (**A**) Patient 3, scans before and after surgery of tumour relapse as well as after the second, fourth and sixth THC cycle (weeks 4, 18 and 29, respectively). (**B**) Patient 8, scans before and after surgery of tumour relapse as well as after the THC cycle (week 3) and at week 32. (**C**) Patient 5, scans before and after surgery of tumour relapse as well as after the fist THC cycle (week 3) and at week 15.

Patient 3 (Table 1, Figure 2A) had an extremely aggressive recurrent glioblastoma multiforme in the left temporal lobe that was evident shortly after the extensive surgical resection of the primary tumour. The recurrent tumour was marginally removed and a total of six THC cycles was administered. During the first three cycles, tumour growth was curbed for about 9 weeks. As the patient showed a clear improvement of clinical symptoms (e.g. dysphasia and cranial hypertension disappeared and haemiparesis ameliorated), three more cannabinoid cycles were administered. However, the KPS started to decline at week 21.

Patient 8 (Table 1, Figure 2B) had an actively growing recurrent glioblastoma multiforme in the right frontal lobe that was partially resected. One THC cycle was subsequently administered, although in view of the high tolerance of the patient the cycle contained more THC than those administered to other patients (Table 1). Tumour volume did not stabilise and followed a continuous increase, but the patient's clinical symptoms largely improved (e.g. cephalalgia and hallucinations disappeared and motor deficit attenuated). However, the KPS started to decline at week 20.

Patient 5 (Table 1, Figure 2C) was one of the patients who seemed not to respond to THC, at least regarding expected length of survival. The right parietal-lobe recurrent tumour was slightly removed, and after the first THC cycle, tumour volume kept constant for 5 weeks. During that period, haemiparesis improved and the KPS did not decrease, but tumour progression and clinical symptoms rapidly worsened thereafter despite the administration of a second THC cycle.

### THC action on tumour cells

**Figure 3:**
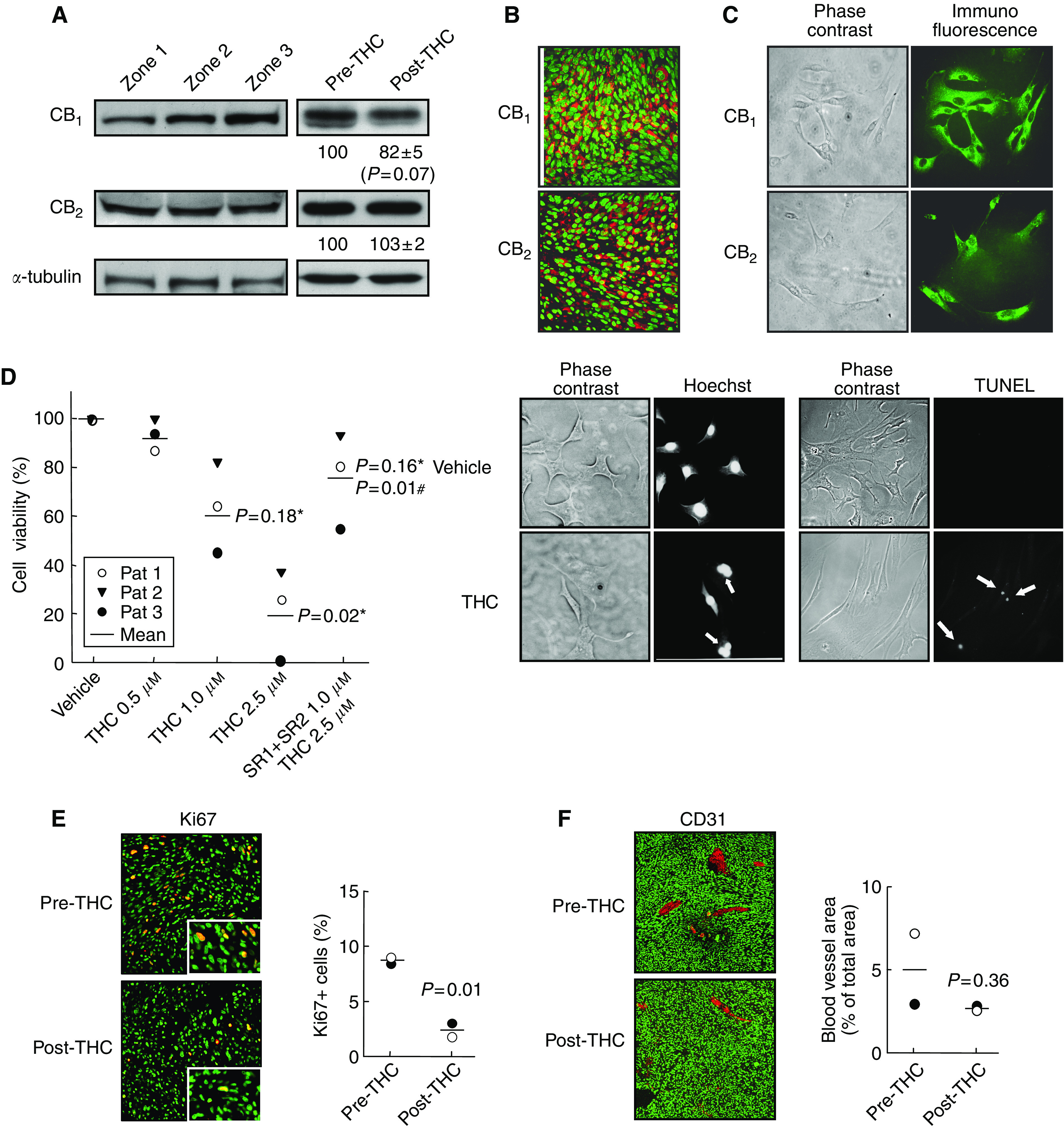
Effect of THC administration on tumour cells. (**A**) Western blot analysis of CB_1_ and CB_2_ receptor expression in three different tumour zones of Patient 1 (left panel) and in tumour biopsies of Patient 1 before and after THC treatment (right panel). Optical density values relative to those of loading controls (*α*-tubulin) are given for Patients 1 and 2 in arbitrary units. (**B**) Immunostaining of CB_1_ and CB_2_ receptors (red) in a tumour biopsy of Patient 1. Nuclei are stained in green. (**C**) Immunostaining of CB_1_ and CB_2_ receptors (green) in tumour cells obtained from Patient 1. (**D**) THC-induced apoptotic death of tumour cells obtained from Patients 1–3. Cells were incubated for 48 h with THC and/or 1.0 *μ*M SR141716 (SR1) plus 1.0 *μ*M SR144528 (SR2). Statistical comparison *vs* vehicle (^*^) or *vs* 2.5 *μ*M THC alone (#) is given. Arrows point to Hoechst-stained fragmented nuclei or to TUNEL-positive nuclei in cells from Patient 1 treated with 2.5 *μ*M THC. (**E**, **F**) Tumour cell proliferation (Ki67 immunostaining, panel E) and tumour vascularisation (CD31 immunostaining, panel F) as determined by confocal microscopy in Patient 1 (○) and Patient 2 (•) before and after THC treatment. Insets in panel E show higher-magnification micrographs. Cell nuclei are stained in green. Representative micrographs of Patient 1 biopsies are shown.

To gain further insight into how THC may affect tumour growth, we determined various cellular parameters in the tumours. The expression of cannabinoid receptors in tumour biopsies was examined by Western blot (Figure 3A) and immunofluorescence (Figure 3B and C). The tumours from the nine patients expressed different amounts of CB_1_ and CB_2_ receptors, but no correlation was found between receptor-type expression and survival (data not shown). Because cannabinoid receptors are known to desensitise upon prolonged occupancy (Howlett *et al*, 2002), it is conceivable that this may hamper the efficacy of long-term treatments. We therefore determined CB receptor expression after THC administration to two patients. Data from Patients 1 and 2 showed a slight decrease in CB_1_ receptor expression and no change in CB_2_ receptor expression (Figure 3A), which might reflect a predominant binding of THC to the former protein or its higher susceptibility to desensitisation.

We next tested the functionality of cannabinoid receptors in the inhibition of tumour cell growth. For this purpose, we isolated tumour cells from glioblastoma biopsies, and observed that THC decreased the number of viable cells in the cultures. This effect relied on CB receptor activation as the CB_1_ antagonist SR141716 together with the CB_2_ antagonist SR144528 prevented cannabinoid action (Figure 3D). THC growth-inhibiting action was due at least in part to apoptosis, as determined by Hoechst 33258 and TUNEL staining (Figure 3D). Likewise, in Patients 1 and 2, THC treatment *in vivo* was associated with reduced tumour-cell proliferation (Ki67 immunostaining) (Figure 3E). Δ^9^-Tetrahydrocannabinol administration tended to decrease tumour vascularisation (CD31 immunostaining) in those two patients, but the effect was not statistically significant (Figure 3F).

## DISCUSSION

Here we report the first clinical study aimed at evaluating cannabinoid antitumoral action. Owing to obvious ethical and legal reasons, this pilot study was conducted in a cohort of terminal patients harbouring actively growing recurrent tumours. Although the use of cannabinoids in medicine may be limited by their well-known psychotropic effects, it is generally believed that cannabinoids display a fair drug safety profile and that their potential adverse effects are within the range of those accepted for other medications, especially in cancer treatment (Guzmán, 2003; Hall *et al*, 2005; Iversen, 2005). In line with this idea, THC delivery in our study was safe and could be achieved without overt psychoactive effects. As the possible antitumoral action of nabilone has never been evaluated in preclinical trials, THC was the unique cannabinoid receptor agonist available for the present human study. Nonetheless, most likely THC is not the most appropriate cannabinoid agonist for future antitumoral strategies owing to its high hydrophobicity, relatively weak agonistic potency and ability to elicit CB_1_-mediated psychoactivity. Unfortunately, the current synthetic cannabinoid agonists that have been reported to exert antitumoral actions in animal models and that could theoretically circumvent – at least in part – the pharmacokinetic and pharmacodynamic limitations of THC (e.g. WIN-55,212-2, a more potent and less hydrophobic CB_1_/CB_2_-mixed agonist (Galve-Roperh *et al*, 2000), and JWH-133, a more potent CB_2_-selective agonist (Sánchez *et al*, 2001)) are still very far from the clinical application owing to the lack of thorough preclinical toxicology studies.

This is not only the first clinical study to assess cannabinoid antitumoral action but also the first human study in which a cannabinoid is administered intracranially. This route of administration was used to mimic our preclinical studies in rodents (Galve-Roperh *et al*, 2000) and has been previously used for the delivery of other cytotoxic drugs such as carmustine to patients with malignant brain tumours (Brem *et al*, 1995). Nonetheless, we note that a non-invasive, less traumatic (e.g. oral) route would be more desirable in the clinical practice. Although intratumoral delivery may allow a high local concentration of the drug *in situ*, in the case of large tumours such as those treated in the present study, the local perfusion through a catheter placed at one point of the tumour constitutes an obvious limitation of the technique. Further studies should assess the distribution pattern of the THC solution within the tumour as well as within the whole brain.

Owing to the characteristics of this study the effect of THC on patient survival was unclear, and an evaluation of survival would require a larger trial with a different design. In this context, pilot placebo-controlled trials for recurrent glioblastoma multiforme with temozolomide, the current benchmark for the management of malignant gliomas, showed a slight impact on overall length of survival (median survival=24 weeks; 6-month survival=46–60%) (Dinnes *et al*, 2002; Nagasubramanian and Dolan, 2003). Likewise, the efficacy of a biodegradable polymer impregnated with carmustine was evaluated in patients with recurrent high-grade gliomas requiring re-operation (Brem *et al*, 1995). The median survival was 31 weeks, but it should be noted that in that study one-third of the treated patients had tumours with better prognosis than glioblastoma multiforme, for example, oligodendrogliomas and anaplastic astrocytomas. Recurrent glioblastoma multiforme is an extremely rapid and lethal disease, and trials in newly diagnosed tumours have allowed a clear improvement in the therapeutic efficacy of temozolomide and carmustine through the development of various administration regimes (Lonardi *et al*, 2005; Stupp *et al*, 2005; Reardon *et al*, 2006). It is therefore conceivable that better outcomes could also be obtained with cannabinoid-based therapies in newly diagnosed gliomas.

Most of the experiments performed so far in animal models of cancer have evidenced a tumour growth-inhibiting action of cannabinoids (Guzmán, 2003). However, a few studies have shown that THC may induce proliferation of tumour cells *in vitro* (Hart *et al*, 2004) and *in vivo* (Zhu *et al*, 2000; McKallip *et al*, 2005). The latter was attributed to a cannabinoid-induced inhibition of host antitumour immunity and was evident in models in which xenografted tumour cells did not express significant levels of cannabinoid receptor, therefore disabling cannabinoid receptor-mediated tumour-cell killing. The present study clearly supports that THC does not facilitate tumour growth nor decreases patient survival, at least in our cohort of brain tumour patients expressing cannabinoid receptors.

In view of the fair safety profile of THC, together with its possible antiproliferative action on tumour cells reported here and in other studies (Guzmán, 2003), it would be desirable that additional trials – on this and other types of tumours – were run to determine whether cannabinoids – as single drugs or in combination with established antitumoral drugs – could be used, other than for their palliative effects, to inhibit tumour growth. In particular, our next goal is to evaluate the efficacy of THC in patients with newly diagnosed gliobastoma multiforme.

## Figures and Tables

**Table 1:** Summary of patient characteristics, treatment and outcome

					**THC treatment**				
**Patient**	**Age (sex)**	**KPS**	**Tumour location**	**Recurrent-tumour volume (cm^3^)**	**Total days**	**Number of cycles**	**Total dose (mg)**	**Time between first and second surgery (wk)**	**Time from second surgery to death (wk)**
1	47 (m)	90	L–O	120	30	2	1.46	70	19
2	58 (m)	80	R–T	69	26	4	1.29	63	18
3	35 (m)	80	L–T	40	64	6	3.29	9	53
4	67 (m)	70	R–T	76	11	1	0.81	23	24
5	51 (f)	70	R–P	41	15	2	1.13	28	17
6	64 (f)	90	R–T	58	10	1	0.80	8	9
7	69 (f)	90	L–T	43	21	3	1.68	112	29
8	51 (f)	90	R–F	52	10	1	1.60	24	49
9	55 (f)	70	L–T	76	11	1	1.28	38	24

Abbreviations: f=female; F=frontal; KPS=Karnofsky performance score; L=left; m=male; O=occipital; P=parietal; R=right; T=temporal; wk=week.

## References

[bib1] Afra D, Baron B, Bonadonna G, Burderr S, Parmar MKB, Stenning SP, Stewart LA, Curran Jr WJ, Green SB, Hildebrand J, Scott CB, Shapiro W, Souhami RL, Thomas D, Trojanowski T, Urtasun RC, Walker MD (2002) Chemotherapy in adult high-grade glioma: a systematic review and meta-analysis of individual patient data from 12 randomised trials. Lancet 359: 1011–1018 11937180 10.1016/s0140-6736(02)08091-1

[bib2] Blázquez C, Casanova ML, Planas A, del Pulgar TG, Villanueva C, Fernández-Aceñero MJ, Aragonés J, Huffman JW, Jorcano JL, Guzmán M (2003) Inhibition of tumor angiogenesis by cannabinoids. FASEB J 17: 529–531 12514108 10.1096/fj.02-0795fje

[bib3] Blázquez C, González-Feria L, lvarez L, Haro A, Casanova ML, Guzmán M (2004) Cannabinoids inhibit the vascular endothelial growth factor pathway in gliomas. Cancer Res 64: 5617–5623 15313899 10.1158/0008-5472.CAN-03-3927

[bib4] Brem H, Piantadosi S, Burger PC, Walker M, Selker R, Vick NA, Black K, Sisti M, Brem S, Mohr G, Muller P, Morawetz R, Schold SC (1995) Placebo-controlled trial of safety and efficacy of intraoperative controlled delivery by biodegradable polymers of chemotherapy for recurrent gliomas. Lancet 345: 1008–1012 7723496 10.1016/s0140-6736(95)90755-6

[bib5] Dinnes J, Cave C, Huang S, Milne R (2002) A rapid and systematic review on the effectiveness of temozolomide for the treatment of recurrent malignant glioma. Br J Cancer 86: 501–505 11870527 10.1038/sj.bjc.6600135 PMC2375282

[bib6] Galve-Roperh I, Sánchez C, Cortés ML, Gómez del Pulgar T, Izquierdo M, Guzmán M (2000) Anti-tumoral action of cannabinoids: involvement of sustained ceramide accumulation and extracellular signal-regulated kinase activation. Nat Med 6: 313–319 10700234 10.1038/73171

[bib7] Gaoni Y, Mechoulam R (1964) Isolation, structure, elucidation and partial synthesis of an active constituent of hashish. J Am Chem Soc 86: 1646–1647

[bib8] Gómez del Pulgar T, de Ceballos ML, Guzmán M, Velasco G (2002) Cannabinoids protect astrocytes from ceramide-induced apoptosis through the phosphatidylinositol 3-kinase/protein kinase B pathway. J Biol Chem 277: 36527–36533 12133838 10.1074/jbc.M205797200

[bib9] Guzmán M (2003) Cannabinoids: potential anticancer agents. Nat Rev Cancer 3: 745–755 14570037 10.1038/nrc1188

[bib10] Hall W, Christie M, Currow D (2005) Cannabinoids and cancer: causation, remediation, and palliation. Lancet Oncol 6: 35–42 15629274 10.1016/S1470-2045(04)01711-5

[bib11] Hart S, Fischer OM, Ullrich A (2004) Cannabinoids induce cancer cell proliferation via tumor necrosis factor *α*-converting enzyme (TACE/ADAM17)-mediated transactivation of the epidermal growth factor receptor. Cancer Res 63: 1943–1950 10.1158/0008-5472.can-03-3720 15026328

[bib12] Howlett AC, Barth F, Bonner TI, Cabral G, Casellas P, Devane WA, Felder CC, Herkenham M, Mackie K, Martin BR, Mechoulam R, Pertwee RG (2002) International Union of Pharmacology. XXVII. Classification of cannabinoid receptors. Pharmacol Rev 54: 161–202 12037135 10.1124/pr.54.2.161

[bib13] Iversen L (2005) Long-term effects of exposure to cannabis. Curr Opin Pharmacol 5: 69–72 15661628 10.1016/j.coph.2004.08.010

[bib14] Kleihues P, Louis DN, Scheithauer BW, Rorke LB, Reifenberger G, Burger PC, Cavenee WK (2002) The WHO classification of tumors of the nervous system. J Neuropathol Exp Neurol 61: 215–225 11895036 10.1093/jnen/61.3.215

[bib15] Lonardi S, Tosoni A, Brandes AA (2005) Adjuvant chemotherapy in the treatment of high grade gliomas. Cancer Treat Rev 31: 79–89 15847978 10.1016/j.ctrv.2004.12.005

[bib16] McKallip RJ, Nagarkatti M, Nagarkatti PS (2005) Delta-9-tetrahydrocannabinol enhances breast cancer growth and metastasis by suppression of the antitumor immune response. J Immunol 174: 3281–3289 15749859 10.4049/jimmunol.174.6.3281

[bib17] Mechoulam R, Hanus L (2000) A historical overview of chemical research on cannabinoids. Chem Phys Lipids 108: 1–13 11106779 10.1016/s0009-3084(00)00184-5

[bib18] Mechoulam R, Spatz M, Shohami E (2002) Endocannabinoids and neuroprotection. Sci STKE 129: RE5 10.1126/stke.2002.129.re5 11972360

[bib19] Molina-Holgado E, Vela JM, Arevalo-Martín A, Almazán G, Molina-Holgado F, Borrell J, Guaza C (2002) Cannabinoids promote oligodendrocyte progenitor survival: involvement of cannabinoid receptors and phosphatidylinositol 3-kinase/Akt signaling. J Neurosci 22: 9742–9753 12427829 10.1523/JNEUROSCI.22-22-09742.2002 PMC6757831

[bib20] Munson AE, Harris LS, Friedman MA, Dewey WL, Carchman RA (1975) Antineoplastic activity of cannabinoids. J Natl Cancer Inst 55: 597–602 1159836 10.1093/jnci/55.3.597

[bib21] Nagasubramanian R, Dolan ME (2003) Temozolomide: realizing the promise and potential. Curr Opin Oncol 15: 412–418 14624222 10.1097/00001622-200311000-00002

[bib22] Piomelli D (2003) The molecular logic of endocannabinoid signalling. Nat Rev Neurosci 4: 873–884 14595399 10.1038/nrn1247

[bib23] Reardon DA, Rich JN, Friedman HS, Bigner DD (2006) Recent advances in the treatment of malignant astrocytoma. J Clin Oncol 24: 1253–1265 16525180 10.1200/JCO.2005.04.5302

[bib24] Sánchez C, de Ceballos ML, Gómez del Pulgar T, Rueda D, Corbacho C, Velasco G, Galve-Roperh I, Huffman JW, Ramon y Cajal S, Guzmán M (2001) Inhibition of glioma growth *in vivo* by selective activation of the CB_2_ cannabinoid receptor. Cancer Res 61: 5784–5789 11479216

[bib25] Stupp R, van den Bent MJ, Hegi ME (2005) Optimal role of temozolomide in the treatment of malignant gliomas. Curr Neurol Neurosci Rep 5: 198–206 15865885 10.1007/s11910-005-0047-7

[bib26] Yamanaka R (2006) Novel immunotherapeutic approaches to glioma. Curr Opin Mol Ther 8: 46–51 16506525

[bib27] Zhu LX, Sharma S, Stolina M, Gardner B, Roth MD, Tashkin DP, Dubinett SM (2000) 9-Tetrahydrocannabinol inhibits antitumor immunity by a CB2 receptor-mediated, cytokine-dependent pathway. J Immunol 165: 373–380 10861074 10.4049/jimmunol.165.1.373

